# The Ecology of Defensive Medicine and Malpractice Litigation

**DOI:** 10.1371/journal.pone.0150523

**Published:** 2016-03-16

**Authors:** Angelo Antoci, Alessandro Fiori Maccioni, Paolo Russu

**Affiliations:** Department of Economics and Management, University of Sassari, Sassari, Italy; Middlesex University London, UNITED KINGDOM

## Abstract

Using an evolutionary game, we show that patients and physicians can interact with predator-prey relationships. Litigious patients who seek compensation are the ‘predators’ and physicians are their ‘prey’. Physicians can adapt to the risk of being sued by performing defensive medicine. We find that improvements in clinical safety can increase the share of litigious patients and leave unchanged the share of physicians who perform defensive medicine. This paradoxical result is consistent with increasing trends in malpractice claims in spite of safety improvements, observed for example in empirical studies on anesthesiologists. Perfect cooperation with neither defensive nor litigious behaviors can be the Pareto-optimal solution when it is not a Nash equilibrium, so maximizing social welfare may require government intervention.

## Introduction

Medical malpractice litigation may be as old as medicine itself. However, it only became the focus of economic research in the early 1970s, when the cost of malpractice insurance reached record highs because of commensurate increases in lawsuits. Defensive medicine is the practice performed by health care providers to safeguard themselves from patients' claims, while disregarding improvements in patients' health [1,2]. Through defensive medicine, physicians can discourage patients from suing and minimize their chance of being held liable in the event of lawsuits. It can take the form of avoidance behavior and is called *negative* defensive medicine when the physician refuses to perform high risk procedures. It can also take the form of assurance behavior and is called *positive* defensive medicine when it is performed using extra tests or procedures. Positive defensive medicine, which we study in this paper, leads to unnecessary diagnostic and therapeutic interventions, which may be invasive and costly. Theoretical research often considers the inefficient provision of medical services as a principal-agent problem and describes its market failures as being due to asymmetric information, moral hazard and conflicts of interest [3–7]. The literature generally agrees that physicians’ behavior does not perfectly fit the neoclassical theory of firms, because of the following aspects [8]. Physicians tend to maximize their profits, but they may also give up some income to promote patients' welfare. Such conduct is consistent with the income/leisure tradeoff that determines supply in labor microeconomics and with altruistic behavior observed in economic experiments [9–11], even in extreme forms [12,13]. Physicians can set the quantity of medical treatment, which is not directly contractible, in partial response to self-interest and subject to demand constraints proportional to the benefit of patients. Physicians can encourage unnecessary health care by increasing their observable effort when treating insured patients [14], or by increasing their unobservable effort and observable care to prevent patients from switching to a competitor in case of adverse events [15]. This latter over-treatment can be considered a contingent form of positive defensive medicine. Superfluous but profitable therapies are more likely when physicians are less fearful of liability [16]. As regards negative defensive medicine, maximizing profits can also induce physicians to under-provide services to the high severity patient if they face liability [17–20]. Physicians can also perform defensive medicine because of fear of reputational losses [21,22]. Stricter negligence standards can lead to more defensive but less negligent medicine, which may increase social welfare [23], although this possibility is controversial [24].

Defensive medical practices are widespread, particularly in specialties at high risk of litigation, such as surgery, obstetrics and gynecology [25–28]. Throughout their career, U.S. surgeons will almost certainly face a malpractice claim, while there is a 70% probability of their facing an indemnity payment [28]. The liability system influences defensive medical practices [2,16] and the costs of medical malpractice insurance [29], but the impact of legal reforms is still disputed [30–32]. Assessing the economic impact of the medical liability system (including defensive medicine) is notoriously difficult because of the lack of reliable evidence and, therefore, its cost estimates vary widely, from 2% to 10% of health care spending in the U.S. [33,34]. The frequency of malpractice claims increased at nearly 10% a year in the 1970s and 1980s [35,36]; since then, it has been moderately stable [28,37]. The factors that explain this increase in claims are not yet fully understood [36]. Empirical data suggest a paradoxical positive relationship between clinical safety and litigation rates. Anesthesiology provides a clear example. In the mid-1980s, this specialty achieved impressive improvements in safety through technological advances and the diffusion of monitoring standards [38,39]. However, empirical studies reveal an increase between 1980 and 1997 in malpractice claims against U.S. and Canadian anesthesiologists [40,41]. In that period, despite a nearly tenfold decrease in the anesthesia mortality rate [38,39], the claims for anesthesia-related death in the U.S. had barely declined [41]. This different pace of change suggests that survival improvements were followed by an increase in litigiousness.

We explain the complex (and somewhat paradoxical) interactions between defensive medicine, malpractice litigation and clinical risk by means of evolutionary game theory [42–45]. To our knowledge, a similar approach is missing from the related literature. We propose an original ‘defensive medicine game’ that describes, in an evolutionary context, defensive and litigious behaviors of boundedly rational individuals [42]. The game works as follows. When adverse events occur, patients can choose whether to pursue litigation against their physician. Conversely, physicians can choose whether to practice defensive medicine to prevent negligence charges. We assume that the shares of defensive physicians and litigious patients in their respective populations follow the replicator dynamics. When a mixed-strategy Nash equilibrium exists, patients and physicians have predator-prey relationships of the Lotka-Volterra type. Litigious patients can be seen as predators, and physicians as their preys; defensive physicians, meanwhile, are adapted prey who have improved their fitness through mutation. We show that, via natural selection, improvements in clinical safety can increase the equilibrium share of litigious patients, while leaving unchanged the equilibrium share of defensive physicians. This paradoxical result is consistent with the increasing trends in malpractice claims despite safety improvements, observed in empirical studies [39].

Finally, we show that perfect cooperation between physicians and patients can be the social optimum under the necessary, but not sufficient, condition that defensive medicine has no direct benefit to patients. If this condition holds, then the mixed-strategy Nash equilibrium, when it exists, is always *less* efficient (in the sense of Pareto) than perfect cooperation. Therefore, there can be a tension between maximizing the individual’s own payoff and maximizing the social welfare, which is a feature typical of social dilemmas [46–48]. Maximizing welfare through perfect cooperation requires government intervention. Policies for this purpose may draw on the experience of Sweden and New Zealand [49,50]. In these countries, patients are compensated by the government for preventable unexpected injuries, while physicians are not financially liable for treatment-related adverse events (however, they may face disciplinary action from their professional body).

## Model and Methods

We propose an evolutionary game between a population of physicians and a population of patients. In each instant of time *t*∈[0,+∞), there is a large number of random pairwise encounters between physicians and patients. In each encounter, a physician provides a risky medical treatment to a patient. An adverse event can occur during the treatment with probability *p*∈(0,1) or not occur with probability 1−*p*. If an adverse event occurs, the patient suffers a damage *R*>0 and can choose, at a cost *C*_*L*_>0, to sue the physician for medical malpractice. If winning the malpractice litigation, the patient would get full compensation *R* from the losing physician. If losing the litigation, the patient would pay *K*>0 to the winning physician as reparation for legal and reputation losses. We assume, with no loss of generality, that the compensation *R* received by the patient is equal to the damage suffered, because this assumption appears to have no influence on the system dynamics and on the welfare results.

The outcome of the litigation is uncertain and depends on the kind of medical care previously provided by the physician. Physicians can either practice defensive medicine or not. The latter choice costs the physician *C*_*ND*_≥0. Practicing defensive medicine costs the physician a greater amount *C*_*D*_>*C*_*ND*_, it causes the patient a harm *H*≥0 (or a benefit, if *H*<0), and it keeps unchanged the probability *p* that an adverse event occurs. In the event of litigation, if the physician did not practice defensive medicine, the patient wins (and the physician loses) with probability *q*_*ND*_∈(0,1), while the physician wins (and the patient loses) with probability 1−*q*_*ND*_. Conversely, if the physician practiced defensive medicine, the patient wins (and the physician loses) with probability *q*_*D*_∈(0,1), while the physician wins (and the patient loses) with probability 1−*q*_*D*_. We assume *q*_*D*_<*q*_*ND*_; that is, defensive medicine protects physicians by decreasing their probability of losing an eventual litigation.

The expected settlement of the litigation is (respectively, when the physician practiced defensive medicine or not):

$$E_{D}=Rq_{D}-K\left(1-q_{D}\right)$$

$$E_{ND}=Rq_{ND}-K\left(1-q_{ND}\right)$$

with *E*_*D*_<*E*_*ND*_. From the physician’s perspective, the terms *E*_*D*_ and *E*_*ND*_ represent the additional expected costs of being sued. Conversely, from the patient’s perspective, *E*_*D*_ and *E*_*ND*_ represent the additional expected benefits of the litigious strategy (with respect to the not-litigious one).

The one-shot game works as follows. The physician can play two pure strategies, *D* or *ND*, respectively representing whether defensive medicine is practiced or not. The patient can play two pure strategies, *L* or *NL*, respectively representing whether to litigate or not in case that an adverse event occurs. Each player chooses the strategy without knowing *ex ante* the other player’s choice.

The physician’s payoffs of strategies *D* and *ND* are:

$$\begin{array}{ccc} \; & \;L & \;NL\\ D & \;\pi_{D}^{L}=-C_{D}-pE_{D} & \;\pi_{D}^{NL}=-C_{D}\\ \; & \; & \;\\ ND & \;\pi_{ND}^{L}=-C_{ND}-pE_{ND} & \;\pi_{ND}^{NL}=-C_{ND} \end{array}$$ (1)

The patient’s payoffs of strategies *L* and *NL* are:

$$\begin{array}{ccc} \; & \;D & \;ND\\ L & \;\pi_{L}^{D}=-H-p\left(R+C_{L}-E_{D}\right) & \;\pi_{L}^{ND}=-p\left(R+C_{L}-E_{ND}\right)\\ \; & \; & \;\\ NL & \;\pi_{NL}^{D}=-H-pR & \;\pi_{NL}^{ND}=-pR \end{array}$$ (2)

The parameters in the payoff matrices satisfy the following conditions: *p*∈(0,1), *R*, *C*_*L*_>0; *C*_*D*_>*C*_*ND*_≥0; *E*_*ND*_>*E*_*D*_. We place no restriction on the sign of the parameter *H*, which will represent a harm for *H*≥0 or a benefit for *H*<0.

Starting from the payoff matrices (1) and (2), we define the evolutionary game as follows. In each instant of time *t*∈[0,+∞), a large number of physicians and patients are randomly paired and play the one-shot game described above. Let *d*(*t*)∈[0,1] represent the share of physicians playing strategy *D* in their total population, and let *l*(*t*)∈[0,1] represent the share of patients playing strategy *L* in their total population, at time *t*. Consequently, 1−*d*(*t*) and 1−*l*(*t*) represent the shares of physicians playing strategy *ND* and of patients playing strategy *NL*, respectively.

The physicians’ expected payoffs from playing strategies *D* and *ND* are, by matrix (1):

$$\Pi_{D}(l)=l\;\pi_{D}^{L}+\left(1-l\right)\;\pi_{D}^{NL}$$

$$\Pi_{ND}(l)=l\;\pi_{ND}^{L}+\left(1-l\right)\;\pi_{ND}^{NL}$$

where *l* and 1−*l* represent the probabilities that a physician is matched with a patient who respectively plays strategy *L* or *NL*.

The patients’ expected payoffs from playing strategies *L* and *NL* are, by matrix (2):

$$\Pi_{L}(d)=d\;\pi_{L}^{D}+\left(1-d\right)\;\pi_{L}^{ND}$$

$$\Pi_{NL}(d)=d\;\pi_{NL}^{D}+\left(1-d\right)\;\pi_{NL}^{ND}$$

where *d* and 1−*d* represent the probabilities that a patient is matched with a physician who plays, respectively, strategy *D* or *ND*.

The average payoffs in the populations of physicians and patients are respectively:

$$\Pi_{PH}=d\;\Pi_{D}(l)+\left(1-d\right)\Pi_{ND}(l)$$

$$\Pi_{PA}=l\;\Pi_{L}(d)+\left(1-l\right)\Pi_{NL}(d)$$

We assume that the time evolution of *d* and *l* is described by the standard replicator dynamics [43–45], a learning-by-imitation model of evolution widely used in economics. The replicator dynamics postulate that players are boundedly rational and update their choices by adopting the relatively more rewarding behavior that emerges from available observations of others’ behaviors. The growth or decline in the adoption rate of a strategy will be proportional to the difference between its payoff and the population average payoff. Accordingly, in the defensive medicine game, the dynamic system is:

$$\begin{array}{lllll} \dot{d} & \;=\; & d\left[\Pi_{D}(l)-\Pi_{PH}\right] & \;=\; & d(1-d)\left[\Pi_{D}(l)-\Pi_{ND}(l)\right]\\ \begin{array}{l} \;\\ \dot{l} \end{array} & \begin{array}{l} \;\\ \;=\; \end{array} & \begin{array}{l} \;\\ l\left[\Pi_{L}(d)-\Pi_{PA}\right] \end{array} & \begin{array}{l} \;\\ \;=\; \end{array} & \begin{array}{l} \;\\ l(1-l)\left[\Pi_{L}(d)-\Pi_{NL}(d)\right] \end{array} \end{array}$$ (3)

where $\dot{d}$ and $\dot{l}$ represent the time derivatives of the shares *d* and *l*, respectively. The factors *d*(1−*d*) and *l*(1−*l*) are always non-negative, so the signs of $\dot{d}$ and $\dot{l}$ will respectively depend on the signs of the payoff differentials:

$$\begin{array}{ccc} \Pi_{D}(l)-\Pi_{ND}(l) & \;=\; & p\;l\;\left(q_{ND}-q_{D}\right)\left(R+K\right)-C_{D}+C_{ND}\\ \; & \;=\; & p\;l\;\left(E_{ND}-E_{D}\right)-C_{D}+C_{ND} \end{array}$$ (4)

$$\begin{array}{ccc} \Pi_{L}(d)-\Pi_{NL}(d) & \;=\; & p\;\left\{\left(R+K\right)\left[\left(q_{D}-q_{ND}\right)d+q_{ND}\right]-K-C_{L}\right\}\\ \; & \;=\; & p\;\left[\left(E_{D}-E_{ND}\right)d+E_{ND}-C_{L}\right] \end{array}$$ (5)

The preceding equations can be derived from the payoff matrices (1) and (2), remembering the definitions of *E*_*D*_ and *E*_*ND*_. The payoff differential of physicians in Eq (4) is an increasing function of *l*, meaning that the relative performance of defensive strategy *D* (with respect to that of strategy *ND*) improves when the population of patients becomes more litigious. Conversely, the payoff differential of patients in Eq (5) is a decreasing function of *d*, meaning that the relative performance of litigious strategy *L* (with respect to that of strategy *NL*) worsens when the population of physicians becomes more defensive.

## Results

The system (3) is defined in the unit square *S* = {(*d*,*l*) ∈ [0,1]^2^}. All sides of this square are invariant; namely, if the pair (*d*,*l*) initially lies on one side, then the whole correspondent trajectory also lies on that side.

Eqs (3) and (4) imply that $\dot{d}=0$ if either *d* = 0,1 or:

$$l=l^{*}\;:=\frac{C_{D}-C_{ND}}{p\left(q_{ND}-q_{D}\right)(R+K)}=\frac{C_{D}-C_{ND}}{p\left(E_{ND}-E_{D}\right)}$$ (6)

where l* > 0 always, and l* < 1 if:

$$C_{D}-C_{ND}<p\left(q_{ND}-q_{D}\right)\left(R+K\right)=p\left(E_{ND}-E_{D}\right)$$ (7)

Furthermore, it results that $\dot{d}>0$ for *l*>l*, and that $\dot{d}<0$ for *l*<l*. The term *C*_*D*_−*C*_*ND*_, on the left in inequality (7), represents the physician’s additional cost of the defensive strategy (with respect to the not-defensive one). Conversely, the term *p*(*E*_*ND*_−*E*_*D*_), on the right in inequality (7), represents the physician’s additional expected benefit of the defensive strategy when played against a litigious patient. Note that the additional expected benefit of the defensive strategy is equal to zero when played against a not-litigious patient.

Analogously, Eqs (3) and (5) imply that $\dot{l}=0$ if either *l* = 0,1 or:

$$d=d^{*}\;:=\frac{R\;q_{ND}-K\left(1-q_{ND}\right)-C_{L}}{\left(q_{ND}-q_{D}\right)\left(R+K\right)}=\frac{E_{ND}-C_{L}}{E_{ND}-E_{D}}$$ (8)

where d*>0 holds if:

$$C_{L}<R\;q_{ND}-K\left(1-q_{ND}\right)=E_{ND}$$ (9)

and d* < 1 holds if:

$$C_{L}>R\;q_{D}-K\left(1-q_{D}\right)=E_{D}$$ (10)

It also results that $\dot{l}>0$ for *d*<d*, and that $\dot{l}<0$ for *d*>d*. The term *C*_*L*_, on the left in inequalities (9) and (10), represents the (additional) cost of the litigious strategy (with respect to the not-litigious one, whose cost is zero). Conversely, the terms *E*_*D*_ and *E*_*ND*_, on the right in inequalities (9) or (10), represent the patient’s additional expected benefit of the litigious strategy (with respect to the not-litigious one) when played, respectively, against a defensive and a not-defensive physician.

According to the above considerations, the four vertices (*d*,*l*) = (0,0),(1,0),(0,1),(1,1) of the square *S* are always stationary states of the dynamic system (3). In these stationary states, the populations of physicians and patients play only one strategy. In (*d*,*l*) = (1,1) all physicians play *D* and all patients play *L*; in (*d*,*l*) = (0,0) all physicians play *ND* and all patients play *NL*; and so on. Another stationary state of the system (3) is the intersection point (*d*,*l*) = (d*,l*) of the straight lines (6) and (8), if it belongs to the square *S*. If conditions (7), (9) and (10) are satisfied, it results that d*,l*⊰(0,1); therefore, (*d*,*l*) = (d*,l*) belongs to the interior of *S* and all the strategies *D*, *ND*, *L* and *NL* coexist in such a state. Finally, all the points belonging to the side of *S* with *l* = 1 are stationary states in the case where l* = 1 (*i*.*e*., when inequality (7) is satisfied as an equality). Similarly, all the points belonging to the side of *S* with *d* = 0 or *d* = 1 are stationary states if, respectively, d* = 0 or d* = 1 (*i*.*e*., when, respectively, inequality (9) or (10) is satisfied as an equality).

### Taxonomy of Dynamic Regimes

**Fig 1:**
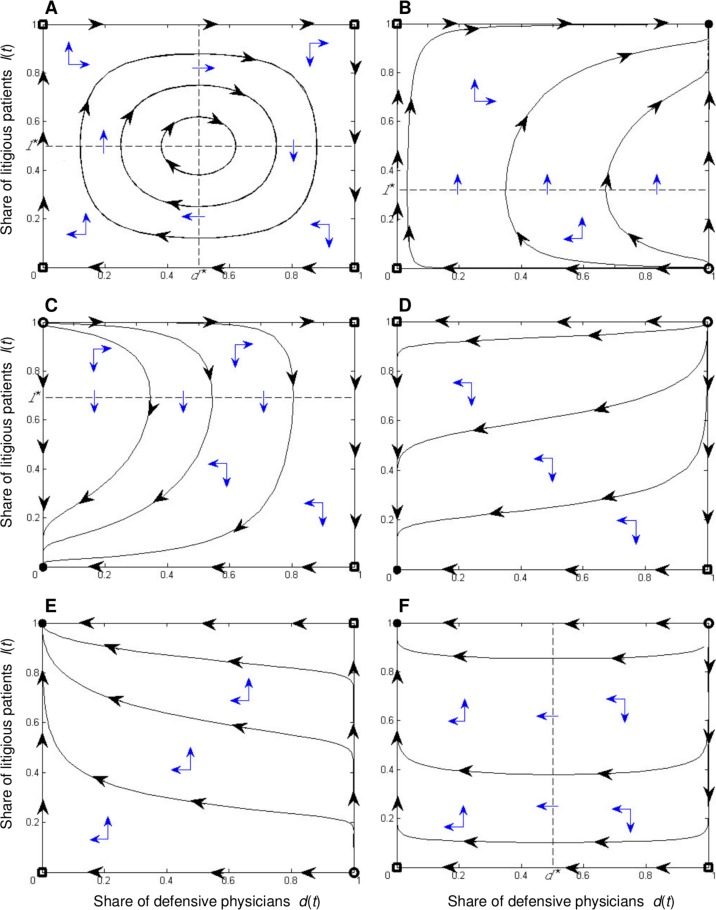
Phase portraits of the replicator dynamics in the defensive medicine game. *Legend*: Continuous lines represent the time evolution of shares of defensive physicians *d*(*t*) and of litigious patients *l*(*t*). Filled dots represent attractors, empty dots represent repellors, and empty squares represent saddle points. Dashed lines d = d* and l = l* are the loci where a sign change occurs in time derivative of the shares *l*(*t*) and *d*(*t*), respectively. The time evolution paths can rotate clockwise around the mixed-strategy Nash equilibrium (*d*,*l*) = (d*,l*) (intersection of dashed lines in panel 1A) or move towards pure-strategy Nash equilibria (attractive stationary states denoted by filled dots in panels 1B−1F).

The dynamics that may be observed by the system (3) have been completely classified [43,44]. We limit our consideration to ‘robust’ dynamic regimes (that is, for brevity, we do not discuss the cases that only occur if equality conditions on parameter values are satisfied). These regimes are illustrated in Fig 1A. The conditions giving rise to each regime are specified in the following propositions.

#### Proposition 1

*If C*_*D*_−*C*_*ND*_<*p*(*E*_*ND*_−*E*_*D*_) *and E*_*D*_<*C*_*L*_<*E*_*ND*_ *hold*, *there exists a (Lyapunov) stable stationary state (d*,l) = (d*,l*) with 0<d*<1 and 0<l*<1, and all the trajectories in the interior of S are closed curves surrounding it (see Fig 1A).

The conditions in the above proposition are equivalent to inequalities (7), (9) and (10), and imply that the game has no dominant strategies. Indeed, the physicians’ best responses result in the following: *to not defend* against the not-litigious patient (because *C*_*D*_>*C*_*ND*_), and *to defend* against the litigious one (by inequality (7)). Similarly, the patients’ best responses result in the following: *to litigate* against the not-defensive physician (by inequality (9)), and *to not litigate* against the defensive one (by inequality (10)).

Fig 1A shows that, when the interior stationary state (*d*,*l*) = (d*,l*) exists, the values *d* and *l* oscillate clockwise around (*d*,*l*) = (d*,l*) for any initial distribution of strategies (*d*_0_,*l*_0_)≠(d*,l*), with *d*_0_,*l*_0_∈(0,1). The initial distribution (*d*,*l*) = (*d*_0_,*l*_0_) will be reached again at the end of every cycle. The trajectories around (*d*,*l*) = (d*,l*) are cyclic because of the signs of the payoff differentials (4) and (5) in each of the four subsets of *S* delimited by the straight lines (6) and (8) (the dashed lines in Fig 1A). The interpretation of such dynamics is simple if we remember that, according to Eq (4), the relative performance of the defensive strategy *D* improves when the population of patients becomes more litigious. Meanwhile, according to Eq (5), the relative performance of the litigious strategy *L* worsens when the population of physicians becomes more defensive. These relations are analogous to predator-prey relations in Lotka-Volterra models, in which litigious patients can be seen as predators and physicians as their prey. *Defensive* physicians, then, can be seen as adapted prey who have improved their fitness through mutation from playing strategy *ND* to playing strategy *D*. The increase in the share of adapted prey (*i*.*e*., defensive physicians) decreases predators’ fitness (*i*.*e*., the payoff differential of litigious patients) and, therefore, causes a decrease in the share of predators (*i*.*e*., litigious patients).

The remaining ‘robust’ regimes that can be observed by the system (3) are described in the following proposition.

#### Proposition 2

*The stationary state that is globally attractive in the interior of the square S is (see inequalities (*7*)*, *(*9*) and (*10*))*:

- (*d*,*l*) = (1,1) *if C*_*D*_−*C*_*ND*_<*p*(*E*_*ND*_−*E*_*D*_) *and C*_*L*_<*E*_*D*_ *hold (see Fig 1B);*
- (*d*,*l*) = (0,0) *if either*:
  - *C*_*D*_−*C*_*ND*_<*p*(*E*_*ND*_−*E*_*D*_) *and C*_*L*_>*E*_*ND*_ *hold (see Fig 1C);*
  - *C*_*D*_−*C*_*ND*_>*p*(*E*_*ND*_−*E*_*D*_) *and C*_*L*_>*E*_*ND*_ *hold (see Fig 1D);*
- (*d*,*l*) = (0,1) *if C*_*D*_−*C*_*ND*_>*p*(*E*_*ND*_−*E*_*D*_) *and C*_*L*_<*E*_*ND*_ *hold (see Fig 1E or 1F when it also holds, respectively, C*_*L*_<*E*_*D*_ *or C*_*L*_>*E*_*D*_*)*.

Propositions 1 and 2 can be verified by standard results in evolutionary game theory [43–45]. Proposition 2 still holds under any evolutionary dynamics that preserve the sign of the time derivatives, such that:

$$sign(\dot{d})=sign\;\left(\Pi_{D}(l)-\Pi_{ND}(l)\right)$$

$$sign(\dot{l})=sign\;\left(\Pi_{L}(d)-\Pi_{NL}(d)\right)$$

in the interior of the square *S*. Conversely, the stability properties of the internal stationary state (*d*,*l*) = (d*,l*) in Proposition 1 may differ if different sign-preserving dynamics are used [44].

### Interpretation of the Dynamics

The interpretation of Propositions 1 and 2 is simple if we keep in mind the meaning of conditions (7), (9) and (10), given, respectively, by:

$$C_{D}-C_{ND}<p\left(E_{ND}-E_{D}\right)$$

$$C_{L}<E_{ND}$$

$$C_{L}>E_{D}$$

Inequality (7) states that the physician’s additional expected benefit of the defensive strategy, when played against a litigious patient, is greater than its additional cost. If this condition holds, the physician’s best responses are *to defend* against litigious patients and *to not defend* against not-litigious patients. Then, the defensive strategy performs better than the not-defensive one (and $\dot{d}>0$ holds) when the share of litigious patients is high enough (*i*.*e*., *l*>l*), while it performs worse than that (and $\dot{d}<0$ holds) when the share of litigious patients is low enough (*i*.*e*., *l*<l*), as in Fig 1A−1C. Note that defending against not-litigious patients is never convenient because it is costly and has zero additional expected benefit. The opposite inequality of (7) implies that the physicians’ dominant strategy is *to not defend* and that the performance of the not-defensive strategy is always better, whatever the share of litigious patients; consequently, $\dot{d}<0$ always holds in the interior of the square *S*, as in Fig 1D−1F.

Inequality (9) states that the patient’s additional cost of the litigious strategy, when played against a not-defensive physician, is less than its additional expected benefit. Similarly, inequality (10) states that the patient’s additional cost of the litigious strategy, when played against a defensive physician, is greater than its additional expected benefit.

If inequality (9) holds, the patient’s best response against a not-defensive physician is *to litigate*. If inequality (10) holds, the patient’s best response against a defensive physician is *to not litigate*. If both (9) and (10) are satisfied, the litigious strategy performs better than the not-litigious one (and $\dot{l}>0$ holds) when the share of defensive physicians is low enough (*i*.*e*., *d*<d*) while it performs worse than that (and $\dot{l}<0$ holds) when the share of defensive physicians is high enough (*i*.*e*., *d*>d*), as in Fig 1A and 1F. Note that, if the opposite inequality of (9) holds, the patients’ dominant strategy is *to not litigate* ($\dot{l}<0$ always holds, see Fig 1C−1D). Conversely, if the opposite inequality of (10) holds, the patients’ dominant strategy is *to litigate* ($\dot{l}>0$ always holds, see Fig 1B and 1E).

### Nash Equilibria

The interior stationary state (*d*,*l*) = (d*,l*) when existing, and the states (*d*,*l*) = (0,0), (0,1), (1,1) when attractive, are Nash equilibria. Consequently, the defensive medicine game always admits a unique Nash equilibrium. This finding follows from standard results in evolutionary game theory [43–45]. We can interpret Nash equilibria as social conventions [51]; that is, as customary and expected states of things in which no single individual has an incentive to modify her choices if the others do not modify theirs. The state (*d*,*l*) = (d*,l*), in which all the available strategies coexist, corresponds to the mixed-strategy Nash equilibrium of the one-shot defensive medicine game. Accordingly, the physician chooses to practice defensive medicine with probability d* and the patient chooses to litigate with probability l*; therefore, (*d*,*l*) = (d*,l*) would represent the equilibrium if all individuals were perfectly rational. We can also interpret (*d*,*l*) = (d*,l*) as the average values of the shares of defensive physicians and litigious patients, over the cycles in Fig 1A. In this sense, (*d*,*l*) = (d*,l*) can estimate the behavior of individuals in random observations over long time periods [44].

### Comparative Statics

Table 1 shows the effects on the mixed-strategy Nash equilibrium (*d*,*l*) = (d*,l*) of changing the parameters of the model. These effects have been estimated from the partial derivatives of the equilibrium coordinates d* and l*. Surprisingly, the equilibrium share of defensive physicians d* does not depend on physicians’ costs *C*_*D*_ and *C*_*ND*_, nor on clinical risk *p*, which still affects the equilibrium share of litigious patients. Conversely, the equilibrium share of litigious patients does not depend on patients’ cost of litigating *C*_*L*_, which still affects the equilibrium share of defensive physicians. These paradoxical results can be explained by the predator-prey relationship between patients and physicians. Accordingly, an evolutionary advantage for a species can be completely offset by a consequent mutation of its competitors. For example, a decrease in clinical risk *p* favors not-defensive physicians and pushes, *ceteris paribus*, the share of defensive physicians below its equilibrium level; therefore, the fitness of litigious patients improves (with respect to not-litigious ones) and their equilibrium share l* permanently increases. When the population of patients becomes more litigious, the fitness of defensive physicians improves (with respect to not-defensive ones) and the share of defensive physicians goes back to its initial equilibrium value d*. Increasing safety in clinical practice, then, may not affect the level of defensive medicine in the long run, but it can increase malpractice litigation against physicians when adverse events occur. Similarly, lowering legal costs *C*_*L*_ will not affect the equilibrium share of litigious patients l*, because the upwards pressure on the share of litigious patients will be contained by a permanent increase in the equilibrium share of defensive physicians d*. Note that a decrease in legal costs *C*_*L*_ may be associated with a reduction in patients’ compensation *R*; for example, when contingency fees give the successful attorneys a percentage of the compensation awarded in favor of their clients, the equilibrium share of litigious patients l* can permanently increase, while that of defensive physicians d* can move either upwards or downwards if, respectively, the change in *C*_*L*_ or *R* is predominant.

**Table 1:** Effects on the mixed-strategy Nash equilibrium (*d*,*l*) = (d*,l*) of an increase in parameters value.

	Effect on equilibrium shares	
Increase in parameter	d*	l*
*p*	↔	↘
*q*_*ND*_	↗	↘
*q*_*D*_	↗	↗
*R*	↗	↘
*K*	↘	↘
*C*_*ND*_	↔	↘
*C*_*D*_	↔	↗
*C*_*L*_	↘	↔
*E*_*ND*_	↗	↘
*E*_*D*_	↗	↗

*Legend*: ↗: increasing; ↘: decreasing; ↔: independent; d*: share of defensive physicians; l*: share of litigious patients; *p*: probability of adverse events during medical treatment; *q*_*D*_, *q*_*ND*_: physician’s probabilities of losing a litigation when, respectively, defending or not; *R*: what a losing physician pays to the litigious patient; *K*: what a not-losing physician receives from the litigious patient; *C*_*D*_, *C*_*ND*_: physician’s costs of, respectively, defending or not; *C*_*L*_: patient’s cost of litigating; *E*_*D*_, *E*_*ND*_: expected settlement of the litigation when, respectively, the physician defends or not. Counter-intuitively, the predator-prey relationship makes l* independent from cost *C*_*L*_, and d* independent from clinical risk *p* and from costs *C*_*ND*_ and *C*_*D*_.

### Welfare Analysis

We compare the stationary states of the game in terms of welfare. A state is more efficient (in the economic sense of Pareto) than another state if, when moving from the former to the latter, the welfare of at least one individual decreases. We measure welfare by means of the population average payoffs Π_*PH*_(*d*,*l*) and Π_*PA*_(*d*,*l*), which correspond, at the stationary states of our dynamic system, to the expected individual payoffs of physicians and patients, respectively. The following two propositions illustrate the most interesting results (see S1 Appendix for proofs).

#### Proposition 3

*When the mixed-strategy Nash equilibrium* (*d*,*l*) = (d*,l*) *exists (see Fig 1A), then* Π_*PH*_(0,0)>Π_*PH*_(d*,l*) *always holds; furthermore*, *it results that* Π_*PA*_(0,0)≥Π_*PA*_(d*,l*) *if H*≥0.

According to Proposition 3, the equilibrium (*d*,*l*) = (d*,l*) can never be more efficient than the non-attractive stationary state with perfect cooperation (*d*,*l*) = (0,0), while (*d*,*l*) = (0,0) is always more efficient than the state (*d*,*l*) = (d*,l*) whenever defensive medicine has no direct benefit to patients (*i*.*e*., when *H*≥0). Remember that, according to the replicator dynamics [44], d* and l* represent the time averages of the shares *d* and *l*, respectively, over the closed trajectories in Fig 1A. Then, Π_*PH*_(d*,l*) and Π_*PA*_(d*,l*) measure the average values along these trajectories of Π_*PH*_(*d*,*l*) and Π_*PA*_(*d*,*l*), respectively. Consequently, the results in Proposition 3 can also be used to compare, in terms of welfare, the stationary state (*d*,*l*) = (0,0) and the closed trajectories around (*d*,*l*) = (d*,l*).

#### Proposition 4

*It results that* Π_*PH*_(0,0)≥Π_*PH*_(1,1) *and* Π_*PA*_(0,0)≥Π_*PA*_(1,1) *if*, *respectively*, *(see inequality (*10*))*:

$$C_{D}-C_{ND}\geq -p\;E_{D}$$ (11)

$$C_{L}\geq E_{D}-\frac{H}{p}$$ (12)

When the stationary state (*d*,*l*) = (1,1) is attractive (see Proposition 2 and Fig 1B), inequality (11) always holds, while inequality (12) holds for high enough ratios *H/p*, because *E*_*D*_ is positive according to Proposition 2; therefore, the state (*d*,*l*) = (0,0) is more efficient than (*d*,*l*) = (1,1) for high enough ratios *H/p* (see S1 Appendix for proof).

Finally, we present an illustrative example of calculation of the *price of anarchy*, which is a measure of how the efficiency of a system degrades due to selfish behavior of its agents [52,53]. For brevity, we consider only the case that arises when the mixed-strategy Nash equilibrium (*d*,*l*) = (d*,l*) exists (see Proposition 1) and is Pareto-dominated by the state of perfect cooperation (*d*,*l*) = (0,0) (see Proposition 3), when the latter is also the social optimum (see S2 Appendix for conditions). In this particular case, the payoffs of all agents are always negative; therefore, the price of anarchy can be computed as the ratio between the social welfare at the Nash equilibrium (*d*,*l*) = (d*,l*) and at the social optimum (*d*,*l*) = (0,0). We measure social welfare by means of the classic *utilitarian* or *Benthamite* social welfare function; that is, as the sum of the population average payoffs of physicians and patients at any given state. These population average payoffs correspond, in both states, to the expected individual payoffs of physicians and patients. Accordingly, the price of anarchy results in the following:

$$PoA=\frac{\Pi_{PH}(d^{*},l^{*})+\Pi_{PA}(d^{*},l^{*})}{\Pi_{PH}(0,0)+\Pi_{PA}(0,0)}=\frac{E_{ND}(C_{D}-C_{ND})+H(E_{ND}-C_{L})}{\left(E_{ND}-E_{D})(C_{ND}+Rp\right)}+1$$

The price of anarchy in the previous equation is negatively correlated to the patient’s costs of litigation *C*_*L*_, because it also results that *H*≥0; therefore, in such a specific scenario, the policy-maker can improve efficiency by raising these legal costs.

## Discussion

This paper has studied the interactions between defensive medicine, malpractice litigation and clinical risk by means of evolutionary game theory. We proposed an original ‘defensive medicine game’ played by physicians and patients, which explains the paradoxical positive relationship between clinical safety and litigation rates observed in empirical studies [40,41]. Using the replicator dynamics, we modelled the time evolution of litigious patients and defensive physicians. We classified the dynamic regimes, found the equilibria and compared the equilibria in terms of welfare and efficiency. The most interesting dynamic regime has a mixed-strategy Nash equilibrium and exhibits predator-prey interactions typical of ecological models. Accordingly, an evolutionary advantage for a species can be completely offset by a consequent mutation of its competitors, with paradoxical consequences. A decrease in clinical risk pushes, *ceteris paribus*, the share of defensive physicians below its equilibrium; in turn, the abundance of vulnerable prey improves the predators’ fitness, so the equilibrium share of litigious patients permanently increases until the share of defensive physicians returns to its initial equilibrium. This interaction can explain why increasing safety in clinical practice may not affect the level of defensive medicine in the long run, but it can increase malpractice litigation against physicians when adverse events occur. Our explanation is complementary to that based on the traditional argument proposed earlier by Mechanic [54]. According to the latter, increasing clinical safety may lead to an increase in patients’ expectations about the quality of health care; if the increase in expectations is too high to meet the actual quality of health care, then patients are more likely to become litigious. We believe that these two explanations can easily coexist. Indeed, our counterintuitive evolutionary findings and the traditional justification based on patients’ unrealistic expectations can both play a role in explaining the lack of negative correlation between clinical safety and patients’ litigiousness.

We concluded our analysis by comparing the possible equilibria of the game in terms of efficiency and welfare. When defensive medicine has no direct benefit to patients, perfect cooperation between physicians and patients is always more efficient than any mixed-strategy Nash equilibrium in which the non-cooperative strategies are partially played. When the harm for patients from defensive medicine is high enough, perfect cooperation is also more efficient than the non-cooperative Nash equilibrium in which everybody litigates or defends. The preceding welfare properties depend crucially on the assumption that defensive medicine is not beneficial for patients (although the population dynamics remain exactly the same regardless of the harmful or beneficial effects of defensive medicine on patients). Conversely, when defensive medicine provides benefit to patients, they can improve their welfare through litigation at the expense of physicians, who should resort to practicing defensive medicine (thus imposing an additional medical effort on them).

The model can be adapted to different concepts of defensive medicine in literature and to other cases of the over-provision of care [2,16]. The relationship between patients and specific health professionals (for example, the family doctor) is often characterized by repeated interactions; this feature can promote altruistic behavior [55] because people tend to internalize others’ perceptions and goals by repeated social contact [56]. Altruism can be introduced in our model in the form of utility interdependence between players, so that the altruist’s utility is a weighted average of her own payoff and of the other player’s payoff [57]. If agents are altruistic, then they will consider (at least to some extent) the effects of their decision on the welfare of the other players. Therefore, cooperation is more likely to occur, even in the absence of coordination. We do not specifically address the ethical and psychological drivers of agents’ behaviors, which can be relevant [22,58].

Our results would be unchanged if we assumed that patients were assisted by lawyers operating under perfect competition conditions. Indeed, a perfectly competitive market would push lawyers’ fees to their marginal cost, and such identical compensation is implicitly included in the model parameters. Also, lawyers paid on a contingency basis might directly replace patients in the game, with minor changes in parameters. Extending the game to lawyers would be suitable to study the impact of information asymmetry on defensive medicine and malpractice settlement costs, which is beyond the scope of this paper.

The possible cyclic dynamics of our model may suggest additional explanations to the observed fluctuations in prices of malpractice insurance [59], which are also attributable to underwriting cycles in the insurance market [60].

The policy implications of our study are various. First, policy-makers should consider the overall underlying dynamics of defensive medicine and malpractice litigation rather than their irregular (and sometimes misleading) short-term trends. Second, because of the predator-prey relationship, clinical advances and legal reforms can have unexpected long-term consequences on the frequency of defensive medicine and malpractice claims. Third, increasing safety in clinical practice can increase the risk for physicians of being sued by patients when accidents occur. Fourth, perfect cooperation can be the social optimum when it is not a Nash equilibrium; therefore, government intervention is needed to maximize social welfare. Policies for this purpose may draw on the experience of Sweden and New Zealand, where physicians are not financially liable for treatment-related adverse events [49,50]. However, evaluating optimal policies still requires more research.

## Supporting Information

S1 Appendix Proofs of Propositions 3 and 4. (DOC)

S2 Appendix Conditions for the social optimum. (DOC)

## References

[1] Tancredi LR, Barondess JA. The problem of defensive medicine. Science. 1978; 200 (4344): 879–882. 644329 10.1126/science.644329

[2] Kessler D, McClellan M. Do doctors practice defensive medicine? Q J Econ. 1996; 111 (2): 353–390.

[3] Arrow KJ. Uncertainty and the welfare economics of medical care. Am Econ Rev. 1963; 53 (5): 941–973.

[4] Pauly MV. The economics of moral hazard: comment. Am Econ Rev. 1968; 58 (3): 531–537.

[5] Zeckhauser R. Medical insurance: A case study of the tradeoff between risk spreading and appropriate incentives. J Econ Theory. 1970; 2 (1): 10–26.

[6] Dionne G, Contandriopoulos AP. Doctors and their workshops: a review article. J Health Econ. 1985; 4 (1): 21–33. 10271143 10.1016/0167-6296(85)90021-9

[7] Hammer PJ, Haas-Wilson D, Sage WM. Kenneth Arrow and the changing economics of health care: ‘Why Arrow? Why now?’. J Health Polit Policy Law. 2001; 26 (5): 835–849. 11765267 10.1215/03616878-26-5-835

[8] McGuire TG. Physician agency. In: Frank RG, McGuire RG, Culyer AJ, Newhouse JP editors. Handbook of Health Economics. Vol. 1. Amsterdam: 461−536; 2000.

[9] Brañas-Garza P. Poverty in dictator games: Awakening solidarity. J Econ Behav Organ. 2006; 60: 306–320.

[10] Brañas-Garza P. Promoting helping behavior with framing in dictator games. J Econ Psychol. 2007; 28: 477–486.

[11] Brañas-Garza P, Cobo-Reyes R, Espinosa MP, Jiménez N, Kovářík J, Ponti G. Altruism and social integration. Games Econ Behav. 2010; 69 (2): 249–257.

[12] Crockett M, Kurth-Nelson Z, Siegel JZ, Dayan P, Dolan RJ. Harm to others outweighs harm to self in moral decision making. Proc Natl Acad Sci U S A. 2014; 111 (48): 17320–17325. 10.1073/pnas.1408988111 25404350 PMC4260587

[13] Capraro V. The emergence of hyper-altruistic behaviour in conflictual situations. Sci Rep. 2015; 4: 9916 10.1038/srep09916 25919353 PMC4412081

[14] Ma CTA; McGuire TG. Optimal health insurance and provider payment. Am Econ Rev. 1997; 685–704.

[15] Allard M, Léger PT, Rochaix L. Provider competition in a dynamic setting. J Econ Manag Strategy. 2009; 18 (2): 457–486.

[16] Currie J, MacLeod BW. First do no harm? Tort reform and birth outcomes. Q J Econ. 2008; 123 (2): 795–830.

[17] Ellis RP, McGuire TG. Provider behavior under prospective reimbursement: cost sharing and supply. J Health Econ. 1986; 5 (2): 129–151. 10287223 10.1016/0167-6296(86)90002-0

[18] Ma CTA. Health care payment systems: cost and quality incentives. J Econ Manag Strategy. 1994; 3 (1): 93–112.

[19] Ellis RP. Creaming, skimping and dumping: provider competition on the intensive and extensive margins. J Health Econ. 1998; 17 (5): 537–555. 10185511 10.1016/s0167-6296(97)00042-8

[20] Feess E. Malpractice liability, technology choice and negative defensive medicine. Eur J Health Econ. 2012; 13 (2): 157–167. 10.1007/s10198-010-0294-7 21222014

[21] Quinn R. Medical malpractice insurance: the reputation effect and defensive medicine. J Risk Insur. 1998; 65 (3): 467–484.

[22] Madarász K. Information projection: model and applications. Rev Econ Stud. 2012; 79 (3): 961–985.

[23] Olbrich A. Heterogeneous physicians, lawsuit costs, and the negligence rule. Int Rev Law Econ. 2008; 28 (1): 78–88.

[24] Gal-Or E. Optimal reimbursement and malpractice sharing rules in health care markets. J Regul Econ. 1999; 16 (3): 237–266.

[25] Dubay L, Kaestner R, Waidmann T. The impact of malpractice fears on cesarean section rates. J Health Econ. 1999; 18 (4): 491–522. 10539619 10.1016/s0167-6296(99)00004-1

[26] Dubay L, Kaestner R, Waidmann T. Medical malpractice liability and its effect on prenatal care utilization and infant health. J Health Econ. 2001; 20 (4): 591–611. 11463190 10.1016/s0167-6296(01)00082-0

[27] Studdert DM, Mello MM, Sage WM, DesRoches CM, Peugh J, Zapert K, et al Defensive medicine among high-risk specialist physicians in a volatile malpractice environment. JAMA. 2005; 293 (21): 2609–2617. 15928282 10.1001/jama.293.21.2609

[28] Jena AB, Seabury S, Lakdawalla D, Chandra A. Malpractice risk according to physician specialty. N Engl J Med. 2011; 365 (7): 629–636. 10.1056/NEJMsa1012370 21848463 PMC3204310

[29] Danzon PM, Epstein AJ, Johnson SJ. The “crisis” in medical malpractice insurance. Brookings-Wharton Pap. Finan. Services. 2004; 1: 55–96.

[30] Sloan FA, Shadle JH. Is there empirical evidence for defensive medicine? A reassessment. J Health Econ. 2009; 28 (2): 481–491. 10.1016/j.jhealeco.2008.12.006 19201500

[31] Mello MM, Kachalia A. Evaluation of options for medical malpractice system reform. Washington, DC: MedPAC Report 10–02; 2010. Available: www.medpac.gov/documents/Apr10_MedicalMalpractice_CONTRACTOR.pdf.

[32] Avraham R, Schanzenbach M. The impact of tort reform on private health insurance coverage. Am. L. & Econ. Rev.. 2010; 12 (2): 319–355.

[33] PriceWaterhouseCoopers. The Factors Fueling Rising Healthcare Costs. 2006. Available: http://www.pwc.com/he_il/il/publications/assets/4the_factors_fueling.pdf.

[34] Mello MM, Chandra A, Gawande AA, Studdert DM. National costs of the medical liability system. Health Aff. 2010; 29 (9): 1569–1577. 10.1377/hlthaff.2009.0807 PMC3048809 20820010

[35] U.S. General Accounting Office. Medical Malpractice: Six State Case Studies Show Claims and Insurance Costs Still Rise Despite Reforms. Washington, DC: GAO/HRD-87-21; 1986.

[36] Danzon PM. Liability for medical malpractice. J Econ Perspect. 1991; 5 (3): 51–69.

[37] Black B, Silver C, Hyman DA, Sage WM. Stability, not crisis: medical malpractice claim outcomes in Texas, 1988−2002. J Empir Leg Stud. 2005; 2: 207–259.

[38] Eichhorn JH. Prevention of intraoperative anesthesia accidents and related severe injury through safety monitoring. Anesthesiology. 1989; 70 (4): 572–577. 2929993 10.1097/00000542-198904000-00002

[39] Kohn LT, Corrigan JM, Donaldson MS, editors. To Err Is Human: Building a Safer Health System. Washington, DC: National Academy Press; 2000. 25077248

[40] Peng PWH, Smedstad KG. Litigation in Canada against anesthesiologists practicing regional anesthesia. A review of closed claims. Can J Anaesth. 2000; 47 (2), 105–112. 10674502 10.1007/BF03018844

[41] Cheney FW, Posner KL, Lee LA, Caplan RA, Domino KB. Trends in anesthesia related death and brain damage: a closed claims analysis. Anesthesiology. 2006; 105: 1081–1086. 17122570 10.1097/00000542-200612000-00007

[42] Maynard Smith J. Evolution and the Theory of Games. Cambridge: Cambridge University Press; 1982.

[43] Hofbauer J, Sigmund K. The Theory of Evolution and Dynamical Systems. Cambridge: Cambridge University Press; 1988.

[44] Weibull JW. Evolutionary Game Theory. MIT press, Cambridge; 1997.

[45] Samuelson L. Evolutionary games and equilibrium selection. Cambridge: Mit Press; 1998.

[46] Kerr N. Motivation losses in small groups: A social dilemma analysis. J Pers Soc Psychol. 1983; 45, 819–828.

[47] Capraro V. A model of human cooperation in social dilemmas. PLoS ONE. 2013; 8 (8): e72427 10.1371/journal.pone.0072427 24009679 PMC3756993

[48] Rand DG., Nowak MA. Human cooperation. Trends Cogn Sci. 2013; 17 (8): 413–425. 10.1016/j.tics.2013.06.003 23856025

[49] Weiler PC. The Case for No-Fault Medical Liability. MD Law Rev. 1993; 52 (4): 908–950.

[50] Towse A, Danzon P. Medical negligence and the NHS: an economic analysis. Health Econ. 1999; 8 (2): 93–101. 10342723 10.1002/(sici)1099-1050(199903)8:2<93::aid-hec419>3.0.co;2-g

[51] Lewis DK. Convention: a Philosophical Study. Cambridge; 1969.

[52] Koutsoupias E, Papadimitriou C. Worst-case equilibria. In: Meinel C, Tison S editors. STACS 99. Berlin: 404−413; 1999.

[53] Papadimitriou C. Algorithms, games, and the internet. Proceedings of the thirty-third annual ACM symposium on Theory of computing. 2001; 749−753.

[54] Mechanic D. Some social aspects of the medical malpractice dilemma. Duke Law J. 1976; 1179–1196.

[55] Rand DG, Ohtsuki H, Nowak MA. Direct reciprocity with costly punishment: Generous tit-for-tat prevails. J Theor Biol. 2009; 256 (1), 45–57. 10.1016/j.jtbi.2008.09.015 18938180 PMC2614626

[56] Goldschmidt W. On the relationship between biology and anthropology. Man (Lond). 1993; 341–359.

[57] Antoci A, Sacco PL, Zarri L. Coexistence of strategies and culturally-specific common knowledge: an evolutionary analysis. J Bioecon. 2004; 6 (2): 165–194.

[58] Vincent C, Phillips A, Young M. Why do people sue doctors? A study of patients and relatives taking legal action. Lancet. 1994; 343 (8913): 1609–1613. 7911925 10.1016/s0140-6736(94)93062-7

[59] Rodwin MA, Chang HJ, Ozaeta MM, Omar RJ. Malpractice premiums in Massachusetts, a high-risk state: 1975 to 2005. Health Aff. 2008; 27 (3): 835–844. 10.1377/hlthaff.27.3.835 18474977

[60] Baker T. Medical malpractice and the insurance underwriting cycle. DePaul Law Review. 2004; 54: 393–438.

