# Supplementary material for: The Ecology of Defensive Medicine and Malpractice Litigation
Source: PLoS One. 2016 Mar 16;11(3):e0150523. doi: 10.1371/journal.pone.0150523 (PMC4794151; doi:10.1371/journal.pone.0150523)
Supplement: S1 Appendix — (DOC) [file pone.0150523.s001.doc]

# S1 Appendix. Proofs of Propositions 3 and 4.

We compare the welfare for the two populations in the states that can be Nash equilibria, (*d*,*l*)=(0,0), (0,1), (1,1) and (*d^*^*,*l^*^*). We measure welfare by means of the population average payoffs Π*_PH_*(*d*,*l*) and Π*_PA_*(*d*,*l*), which correspond (in these states) to the expected individual payoffs of, respectively, physicians and patients.

The values of Π*_PH_*(*d*,*l*) and Π*_PA_*(*d*,*l*) are:

$$\Pi_{P H}(0,0)=\Pi_{ND}(0)=\pi_{N D}^{N L}=-C_{N D}$$

$$\Pi_{P A}(0,0)=\Pi_{NL}(0)=\pi_{N L}^{N D}=-pR$$

$$\Pi_{P H}(0,1)=\Pi_{ND}(1)=\pi_{ND}^{L}=-C_{ND}-pE_{ND}$$

$$\Pi_{P A}(0,1)=\Pi_{L}(0)=\pi_{L}^{ND}=-p\left[R+C_{L}-E_{ND}\right]$$

$$\Pi_{P H}(1,1)=\Pi_{D}(1)=\pi_{D}^{L}=-C_{D}-pE_{D}$$

$$\Pi_{P A}(1,1)=\Pi_{L}(1)=\pi_{L}^{D}=-H-p\left[R+C_{L}-E_{D}\right]$$

$$\Pi_{P H}(d^{*},l^{*})=\Pi_{D}(l^{*})=-C_{D}-l^{*}pE_{D}=\Pi_{ND}(l^{*})=-C_{ND}-l^{*}pE_{ND}$$

$$\Pi_{PA}(d^{*} ,l^{*} )=\Pi_{L}(d^{*} )=-Hd^{*}- p\left[R+C_{L}-E_{ND}+\left(E_{ND}-E_{D}\right)d^{*}\right]=\Pi_{NL}(d^{*})=-Hd^{*}-pR$$

It results Π*_PH_*(0,0)≥Π*_PH_*(*d^*^*,*l^*^*) if:

$$E_{ND}\geq0$$

which always holds (with strict inequality) when (*d*,*l*)=(*d^*^*,*l^*^*) exists, according to Proposition 1 and recalling the definition (9). Furthermore, it results Π*_PA_*(0,0)≥Π*_PA_*(*d^*^*,*l^*^*) if *H*≥0. Thus, the state (*d*,*l*)=(0,0) is more efficient (in the sense of Pareto) than (*d*,*l*)=(*d^*^*,*l^*^*) if *H*≥0, while the state (*d*,*l*)=(*d^*^*,*l^*^*) can never be more efficient than (*d*,*l*)=(0,0).

It results Π*_PH_*(0,0)≥Π*_PH_*(0,1) and Π*_PA_*(0,0)≥Π*_PA_*(0,1) if:

(13) $C_{L}\geq E_{ND}\geq0$

The state (*d*,*l*)=(0,0) is more efficient (in the sense of Pareto) than (*d*,*l*)=(0,1) if inequality (13) holds with at least one strict inequality. The state (*d*,*l*)=(0,1) is never more efficient than (*d*,*l*)=(0,0) because it results *C_L_*>0 and, therefore, the opposite inequality of (13) never holds.

It results Π*_PH_*(0,0)≥Π*_PH_*(1,1) and Π*_PA_*(0,0)≥Π*_PA_*(1,1) if, respectively:

(14) $C_{D}-C_{ND}\geq-p E_{D}$

(15) $C_{L}\geq E_{D}-\frac{H}{p}$

The state (*d*,*l*)=(0,0) is more efficient (in the sense of Pareto) than (*d*,*l*)=(1,1) if inequalities (14) and (15) hold and at least one of them holds with strict inequality; conversely, (*d*,*l*)=(1,1) can be more efficient than (*d*,*l*)=(0,0) if the opposite holds. Note that, if the state (*d*,*l*)=(1,1) is attractive, inequality (14) always holds and inequality (15) holds for high enough ratios *H/p*, because *E_D_* is positive according to Proposition 2 and definition (10); therefore, the state (*d*,*l*)=(0,0) is more efficient than the equilibrium (*d*,*l*)=(1,1) for high enough ratios *H/p*.
