# Supplementary material for: The Ecology of Defensive Medicine and Malpractice Litigation
Source: PLoS One. 2016 Mar 16;11(3):e0150523. doi: 10.1371/journal.pone.0150523 (PMC4794151; doi:10.1371/journal.pone.0150523)
Supplement: S2 Appendix — (DOC) [file pone.0150523.s002.doc]

# S1 Appendix. Conditions for the social optimum.

We illustrate the condition for which the state of perfect cooperation (*d*,*l*)=(0,0) can be a social optimum. We measure the social welfare Π(*d*,*l*) by means of the *utilitarian* (or *Benthamite*) social welfare function, that is, as the sum of the population average payoffs Π*_PH_*(*d*,*l*) and Π*_PA_*(*d*,*l*).

The social welfare at any generic state (*d*,*l*) is:

$$\begin{matrix} \Pi(d,l)=\Pi_{P H}(d,l)+\Pi_{P A}(d,l)=d\left[-C_{D}-lpE_{D}\right]+\left(1-d\right)\left(-C_{ND}-lpE_{ND}\right) \\ +l\left\{-Hd-p\left[R+C_{L}-E_{ND}+\left(E_{ND}-E_{D}\right)d\right]\right\}+\left(1-l\right)\left(-Hd-pR\right) \end{matrix}$$

The social welfare at the state of perfect cooperation (*d*,*l*)=(0,0) is:

$$\Pi(0,0)=\Pi_{PH}(0,0)+\Pi_{PA}(0,0)=-C_{N D}-pR$$

Then, the state of perfect cooperation (*d*,*l*)=(0,0) is a social optimum if it holds Π(0,0)≥Π(*d*,*l*) for all possible values of *d*∈[0,1] and *l*∈[0,1]; some algebraic manipulation reveals that this condition can be always satisfied for sufficiently high levels of harm *H*>0.
